# McSNAC: A software to approximate first-order signaling networks from mass cytometry data

**DOI:** 10.15302/J-QB-022-0308

**Published:** 2023-03

**Authors:** Darren Wethington, Sayak Mukherjee, Jayajit Das

**Affiliations:** 1 Steve and Cindy Rasmussen Institute for Genomic Medicine, Abigail Wexner Research Institute, Nationwide Children’s Hospital, Columbus, Ohio 43205, United States; 2 Biomedical Sciences Graduate Program, College of Medicine, The Ohio State University, Columbus, Ohio 43210, United States; 3 Department of Pediatrics, College of Medicine, The Ohio State University, Columbus, Ohio 43210, United States; 4 Pelotonia Institute for Immuno-Oncology, College of Medicine, The Ohio State University, Columbus, Ohio 43210, United States; 5 Department of Biomedical Informatics, College of Medicine, The Ohio State University, Columbus, Ohio 43210, United States; 6 Biophysics Graduate Program, The Ohio State University, Columbus, Ohio 43210, United States; 7 Battelle Memorial Institute, Columbus, Ohio 43201, United States

**Keywords:** single-cell, CyTOF data, signaling network, kinetics, ODE, McSNAC

## Abstract

**Background:**

Mass cytometry (CyTOF) gives unprecedented opportunity to simultaneously measure up to 40 proteins in single cells, with a theoretical potential to reach 100 proteins. This high-dimensional single-cell information can be very useful in dissecting mechanisms of cellular activity. In particular, measuring abundances of signaling proteins like phospho-proteins can provide detailed information on the dynamics of single-cell signaling processes. However, computational analysis is required to reconstruct such networks with a mechanistic model.

**Methods:**

We propose our Mass cytometry Signaling Network Analysis Code (McSNAC), a new software capable of reconstructing signaling networks and estimating their kinetic parameters from CyTOF data. McSNAC approximates signaling networks as a network of first-order reactions between proteins. This assumption often breaks down as signaling reactions can involve binding and unbinding, enzymatic reactions, and other nonlinear constructions. Furthermore, McSNAC may be limited to approximating indirect interactions between protein species, as cytometry experiments are only able to assay a small fraction of protein species involved in signaling.

**Results:**

We carry out a series of *in silico* experiments here to show (1) McSNAC is capable of accurately estimating the ground-truth model in a scalable manner when given data originating from a first-order system; (2) McSNAC is capable of qualitatively predicting outcomes to perturbations of species abundances in simple second-order reaction models and in a complex *in silico* nonlinear signaling network in which some proteins are unmeasured.

**Conclusions:**

These findings demonstrate that McSNAC can be a valuable screening tool for generating models of signaling networks from time-stamped CyTOF data.

## Author summary

Modeling how cells transfer a signal from an extracellular stimulus to different compartments within the cell is critical to understand how different stimuli result in different cellular responses. We designed a software (McSNAC) that proposes a minimal mathematical model to describe biochemical signaling events learning from time-stamped cytometry data. This software makes linear approximations which hold true in many scenarios, but can also break down under several conditions. We explore this approximation in depth to provide guidelines regarding its applicability. In addition, we provide a user interface for non-technical users to analyze their time-stamped cytometry data with this approach.

## INTRODUCTION

Single cells respond to stimulus *via* receptors that are usually bound to the plasma membrane. These receptors bind to cognate ligands generated by a stimulation, such as viral infection of cells in the local environment, and initiate a series of biochemical signaling reactions leading to activation of many genes that can generate a variety of cell responses such as secretion of specific proteins (*e.g*., cytokines), cell proliferation, or cell death [1–3]. Signaling reactions are composed of biochemical reactions involving a large number of proteins (~ thousands), and it is often challenging to determine key signaling regulators of specific cell responses among a large set of interacting proteins [4,5]. This problem is further confounded as single cells can contain large cell-cell variations of protein abundances where protein abundances are usually distributed broadly (*e.g*., lognormally) in a cell population, even in a clonal population [6]. Since biochemical reaction propensities depend on concentrations of participating molecules, and biochemical reactions are intrinsically stochastic in nature due to thermal fluctuations [7], single cells in clonal cell populations stimulated by the same ligand can show large variations in responses making it difficult to determine key regulators in the face of large variations. Recent advances in single cell cytometry, such as Mass Cytometry, or Cytometry by Time-of-Flight (CyTOF), are currently capable of measuring up to 40 proteins simultaneously in thousands to millions of single cells, with a theoretical limit as high as 100 proteins [8,9]. Thus, measuring signaling protein abundances at different points post stimulation using CyTOF provides time-stamped snapshot data regarding signaling kinetics and cell-cell variation of the kinetics. However, it is difficult to intuitively determine key regulators that give rise to a specific response in a single cell or a group of single cells with this data alone for the following reasons. First, though causal relationships between measured proteins, such as phosphorylation of protein A inducing phosphorylation of protein B, might be known from previous experiments, activation of key regulator proteins can often be produced by multiple proteins interacting synergistically. Second, protein abundances vary widely across cells, and proteins in the same individual cell are not measured across time, thus if specific proteins are activated during signaling kinetics in individual cells remains unknown.

Some of the above challenges are also present in deciphering mechanisms underlying signaling kinetics using population data or traditional flow cytometry data measuring few proteins. Mechanistic mathematical models composed of ordinary differential equations (ODEs) or stochastic models describing biochemical signaling reactions have been used to address these difficulties [10,11]. These models work well when the number of protein species is small and biochemical reaction propensities for reacting proteins are well characterized. However, it is often not the case for newly discovered cell types (*e.g*., myeloid derived suppressor cells) [12] or situations where multiple types of receptors (*e.g*., cytokine receptors and primary cell receptor) synergize [13]. It is challenging to build such mechanistic models in such situations where protein species interacting physically are usually not measured simultaneously and it is unclear what form (*e.g*., first, second or higher order reaction) of reaction propensities should be used for describing the interaction between two protein species separated by many intermediate reactions.

Machine learning has also been employed to characterize ODEs to predict the effects of cellular perturbations [14]. This model is quite useful for mechanistically predicting the effects of perturbations, but does not have a mechanism to incorporate the single-cell resolution offered by CyTOF. It is important to note that signaling processes happen at the single-cell resolution, and thus single-cell experiments contain crucial information on signaling dynamics. This information can be used to identify how cells vary in their protein expressions, and which proteins co-vary with each other in single cells. For these reasons, it is crucial to have a method to form accurate mechanistic models for single-cell signaling networks.

To this end, we developed a first-order mass action reaction kinetics model given by linear ODEs to describe signaling kinetics over a time interval — *e.g*., time between two successive mass cytometry measurements [13,15]. The solution of the model describes the time evolution in a closed form and accounts for cell-cell variations of protein abundances. In this paper we report development of a software package Mass cytometry Signaling Network Analysis Code (McSNAC) that takes flow cytometry data at two successive time points as input, fits the model involving first-order kinetics, and generates parameter estimates for the kinetic rates. The model can then be used for generating predictions regarding changes in protein abundances. We use McSNAC to model synthetic cytometry data to demonstrate: (1) Parameters in the model can be well estimated. (2) Models that include the ground-truth model describing first-order reaction kinetics provide better fits than models without the ground-truth. (3) The first-order models in McSNAC are able to make accurate qualitative predictions to perturbations in protein abundances. (4) The models in McSNAC generate better predictions to perturbations when directionality of the underlying reaction is captured in the model.

### Model

McSNAC describes kinetics of single cell protein abundances using first-order chemical reaction kinetics. Consider *p* number of protein species {*X*_1_, …, *X*_*p*_} measured in cytometry experiments that interact during a signaling process. In McSNAC the abundance *x*^{*α*}^_*i*_ of protein *X*_*i*_ in each cell indexed by *α* changes with time given by the linear ODEs shown below:

(1) $$\frac{dx_{i}^{\left(\alpha \right)}}{dt}=k_{i\to i}x_{i}^{\left(\alpha \right)}-\overset{p}{\underset{j=1}{\sum }}k_{i\to j}x_{i}^{\left(\alpha \right)}+\overset{p}{\underset{j=1}{\sum }}k_{j\to i}x_{j}^{\left(\alpha \right)}$$

where the first-order rate that produces species *X*_*j*_ from *X*_*i*_ is given by *k*_*i*→*j*_ (*k*_*i*→*j*_ ≥ 0 for *i* ≠ *j*). *k*_*i*→*i*_ > 0 denotes self-production or production of *X*_*i*_ from another unmeasured species while *k*_*i*→*i*_ < 0 denotes self-decay. In the above kinetics, the kinetic rates {*k*_*i*→*j*_} are the same across single cells (*i.e*., a *k*_*i*→*j*_ does not depend on the cell index α). This is an approximation as some of these rates could represent nonlinear reaction such as enzymatic modifications where the rates could depend on the abundances of an enzyme which could vary from cell-to-cell. The ODEs in Eq. (1) can be represented in compact form by introducing a *p* × *p* matrix, ***M***, where *M*_*ij*_(*i* ≠ *j*) = *k*_*j*→*i*_, and *M*_*ii*_ = *k*_*i*→*i*_ − ∑_*j*_ *k*_*i*→*j*_, *i.e*.,

(2) $$\frac{dx_{i}^{\left(\alpha \right)}}{dt}=\overset{p}{\underset{j=1}{\sum }}M_{ij}x_{j}^{\left(\alpha \right)}$$

This representation of the mass-action kinetics offers a convenient closed-form solution (details in Materials and Methods section) for $\left\{x_{i}^{\left(\alpha \right)}\left(t_{2}\right)\right\}$ at given $\left\{x_{i}^{\left(\alpha \right)}\left(t_{1}\right)\right\}$, *i.e*., $x_{i}^{\left(\alpha \right)}\left(t_{2}\right)=\sum_{j=1}^{p}{\left[e^{M(t_{2}-t_{1})}\right]}_{ij}x_{j}^{\left(\alpha \right)}\left(t_{1}\right)$.

Mass cytometry experiments are unable to track single cells over time as individual cells are destroyed at the time of measurement. However, it is still possible to follow the kinetics of average values, covariances and higher order moments of the protein abundances that are computed from the snapshot data.

(3) $$\mu_{i}(t)=\frac{1}{N}\overset{N}{\underset{\alpha =1}{\sum }}x_{i}^{\left(\alpha \right)}(t)$$

(4) $$J_{ij}(t)=\frac{1}{N}\overset{N}{\underset{\alpha =1}{\sum }}\left(x_{i}^{\left(\alpha \right)}(t)-\mu_{i}(t)\right)\left(x_{j}^{\left(\alpha \right)}(t)-\mu_{j}(t)\right)$$

The ODEs in Eq. (2) can be solved to relate the average values {*μ*_*i*_} and covariances {*J*_*ij*_} calculated at times *t*_1_ and *t*_2_,

(5) $$\mu_{i}\left(t_{2}\right)=\overset{p}{\underset{j=1}{\sum }}{\left[e^{M\left(t_{2}-t_{1}\right)}\right]}_{ij}\mu_{j}\left(t_{1}\right)$$

(6) $$J\left(t_{2}\right)=e^{M\left(t_{2}-t_{1}\right)}J\left(t_{1}\right)e^{M^{T}\left(t_{2}-t_{1}\right)}$$

The bold form symbols in the above equations denote matrices. These above closed-form solutions make it efficient to compute predicted average values and covariances in the model compared to iteratively solving the ODEs in Eq. (2) numerically and then computing those variables using Eqs. (3) and (4). Matrix exponentials are calculated with the Padé approximation, and require significantly less computational time than standard numerical ODE solution techniques such as Runge-Kutta methods. Given the measured values of {*μ*_*i*_} and {*J*_*ij*_} at time *t*_1_ from cytometry experiments, we estimated the kinetic rates in *M* that best fit cytometry data for {*μ*_*i*_} and {*J*_*ij*_} at a later time *t*_2_. *M* is estimated by minimizing a cost function (Eq. (7)) by employing a simulated annealing algorithm. The cost function is defined as,

(7) $$\chi^{2}=\sum_{i}{\left(1-\frac{\mu {\left(t_{2}\right)}_{i}^{\left(\text{predicted}\right)}}{\mu {\left(t_{2}\right)}_{i}^{\text{(actual})}}\right)}^{2}+\sum_{i,j}{\left(1-\frac{J{\left(t_{2}\right)}^{\left(\text{predicted}\right)}{\;}_{ij}}{J{\left(t_{2}\right)}^{\text{(actual)}}{\;}_{ij}}\right)}^{2}+\sum_{j}{\left(t_{2}-t_{1}\right)}^{2}{\left(\sum_{i}M_{ij}\right)}^{2}$$

Where, *μ*(*t*_2_)^(actual)^ and ***J***(*t*_2_)^(actual)^ are the means and covariances calculated from cytometry data at time *t*_2_ using Eqs. (3) and (4), and, *μ*(*t*_2_)^(predicted)^ and ***J***(*t*_2_)^(predicted)^ are the means and covariances predicted by the model following Eqs. (5) and (6) given *μ*(*t*_1_) and ***J***(*t*_1_) calculated from cytometry data. This cost function has three terms, penalizing different aspects of the fit. The first term penalizes deviations from the observed averages. The second term penalizes deviations from the observed variances and covariances. The third term penalizes kinetics that do not abide by conservation of mass (assuming phospho-groups are transferred and no other changes in abundance occur). In other words, the third term penalizes *k*_*i*→*i*_ ≠ 0 from Eq. (1). If data come from a ground-truth first-order model which abides by conservation of mass, and the ground-truth ***M*** is found, then *χ*^2^ = 0. A more detailed description of this method can be found in Mukherjee *et al*. 2017 [13].

## RESULTS

### McSNAC is capable of accurately estimating first-order reaction rates

Here we describe McSNAC’s ability to (1) model data when the wiring of the ground-truth model is known but the values of the rate constants are unknown; (2) computationally scale as the number of proteins in the data is increased up to 40; and (3) separate models that do or do not include the ground-truth. The synthetic cytometry data were generated for a coupled set of first-order reactions as shown in Reaction (R1) using Eq. (2), where the distributions of {*x*_1_^(*α*)^, …, *x*_*p*_^(*α*)^} at *t* = 0 are chosen from log-normal distributions (see Materials and Methods for details).


(R1) $$X_{1}\overset{k_{1\to 2}}{\to }X_{2}\overset{k_{2\to 3}}{\to }X_{3}\cdots \overset{k_{p-2\to p-1}}{\to }X_{p-1}\overset{k_{p-1\to p}}{\to }X_{p}$$

**Figure 1.**
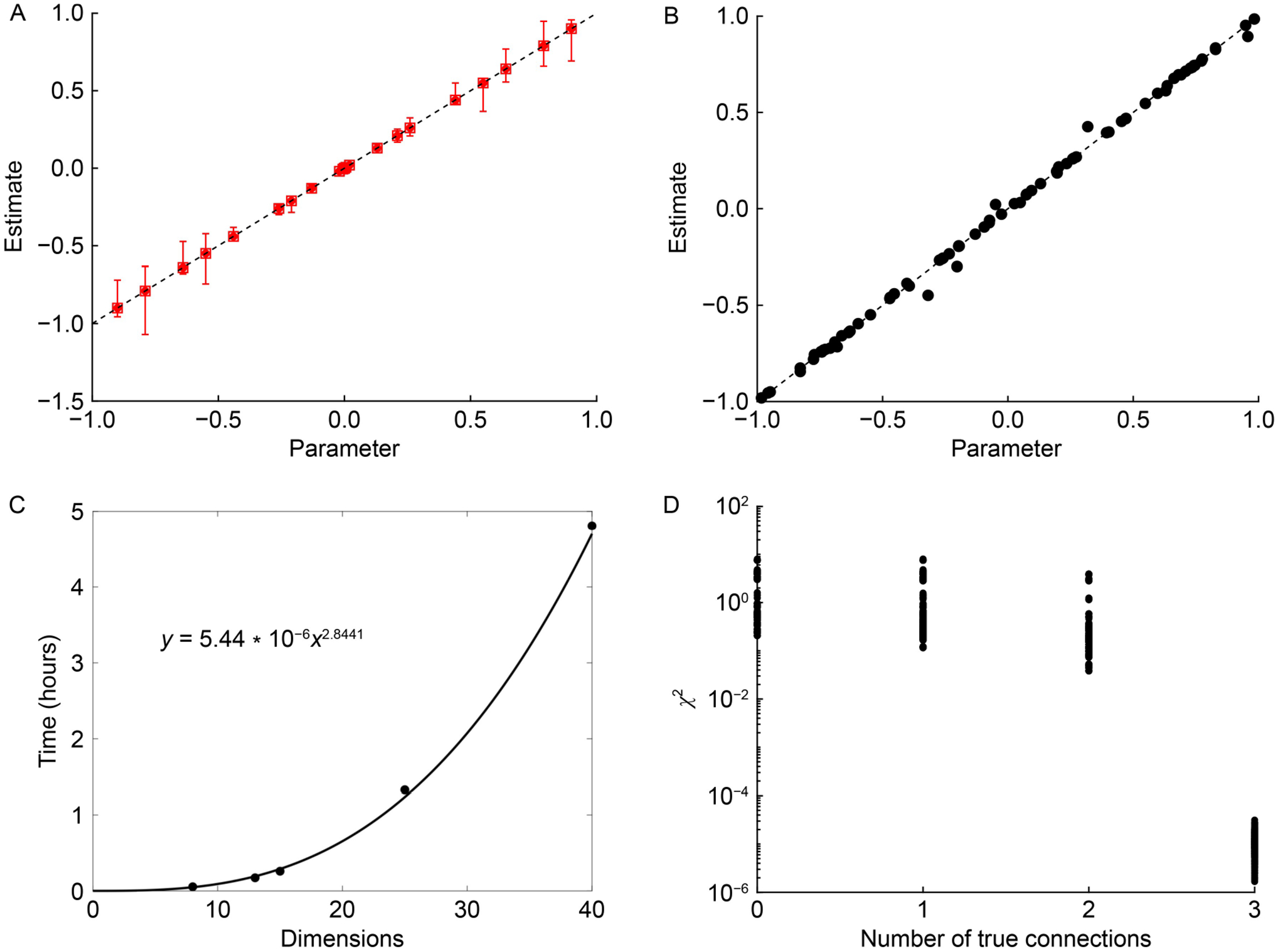
McSNAC is a scalable software capable of reconstructing first-order signaling dynamics. (A) Comparison of manually selected parameters of a 13-dimensional system and the estimated parameter values from McSNAC. Error bars denote confidence intervals on the parameter estimates. The dotted black line represents *y* = *x*, and perfectly estimated parameters will lie on this line. (B) Comparison of manually selected parameters of randomly selected parameters of a 40-dimensional system and the estimated value of each parameter by McSNAC. The dotted black line represents *y* = *x*, and perfectly estimated parameters will lie on this line. (C) The average time (n = 3) required to run McSNAC for the number of dimensions *p* shown for a system represented by Eq. (R1). (D) 2^12^ models were simulated, each one containing either none (0), some (1–2), or all (3) of the *k*_*i*_→_*j*_ connections in the ground-truth model. Models with all (3) of the true connections, comprising the ground-truth model, generate *χ*^*2*^ ≅ 0, indicating a perfect fit.

The rates {*k*_*i*→*j*_} were assigned from a uniform random distribution with a range of 0 to 1, and {*x*_1_^(*α*)^, …, *x*_*p*_^(*α*)^} were computed for 2,500 single cells at two different time points. This constituted the synthetic cytometry data which were generated for a range of *p* between 8 to 40. We applied McSNAC to synthetic data following the architecture in (R1) to determine (1) how accurately parameters {*k*_*i*→*j*_} are estimated; (2) how does the run time increase with increasing *p*. The comparison of the McSNAC estimated parameters and their actual values shows high accuracy in parameter estimation (Fig. 1A–B). The run time of McSNAC on a single CPU (3.0GHz AMD 7302, compiled with *gfortran* with the −*O* optimization flag) ranged from minutes to hours where the case for *p*=40 took 4.8 hours to complete on average (Fig. 1C). Theoretical computational complexity is *O*(*n*^3^), and an empirical fit to the runtimes indicates that this is indeed observed. The complexity is primarily due to matrix multiplication, which means that improvements to runtime are possible with more advanced computational techniques. These results indicate that this technique is scalable with the large number of inputs afforded by CyTOF. It is important to note that exact runtimes vary depending on hyperparameter specification, such as number of temperature cooling iterations and number of Monte Carlo samples per temperature step, and the exact data and model being fitted.

Next, we evaluated the ability of the cost function (Eq. (7)) to separate models that include or do not include the architecture of the ground-truth model. This is relevant for cases where interactions between protein species in a signaling network are not known and several potential network architectures can be hypothesized to describe the data. To test the above property, we generated synthetic cytometry data for a reaction scheme involving four protein species given by *p* = 4 in (R1). If we had no knowledge of the architecture of the ground-truth network that connected the proteins, these four proteins would offer a total of 12 possible *k*_*i*→*j*_’s, and 2^12^ unique network architectures (more details in Materials and Methods section). Applying McSNAC to each of the 2^12^ unique network architectures and determining the goodness-of-fit *χ*^2^ shows that when a model contained the ground-truth architecture it generated close to zero values of *χ*^2^, whereas models that do not include the ground-truth architecture generated larger values of *χ*^2^ (Fig. 1D). These results show that McSNAC is capable of reconstructing the ground-truth signaling dynamics from data resulting from a first-order system, regardless of dimensionality or *a priori* information of its wiring.

In the next sections we show results when McSNAC is applied for data sets that represent scenarios that are commonly present in cytometry datasets studying signaling kinetics. Signaling biochemical reactions contain reactions associated with propensities that are nonlinear functions of abundances of reacting protein species. Moreover, a large number (~ 1,000–10,000) of proteins and their chemically modified forms can be involved in signaling reactions, and CyTOF experiments are able to measure a small fraction of those protein species. Therefore, we tested the ability of the first-order reaction kinetics in McSNAC to approximate the signaling reaction kinetics in the above situations. The following cases are considered: (1) The ground-truth model contains non-linear biochemical reactions, (2) Not all proteins participating in biochemical signaling reactions are measured. We find that McSNAC is able to describe the result of perturbations such as increase and decrease of specific protein abundances in most situations, however, McSNAC performs poorly when it comes to predicting protein abundances at a later time (Supplementary Fig. S1). In the next sections we provide further details regarding McSNACs ability to predict outcomes of perturbations of signaling kinetics due to increase and decrease of specific protein abundances. Such perturbations are routinely performed using specific drugs such as Src family kinase inhibitor, Syk family kinase inhibitors, or small interfering RNA (siRNA).

### McSNAC is capable of approximating simple non-linear systems

We test the ability of McSNAC to capture kinetics in a ground-truth model describing a second-order reaction in which two reactants combine to form a product. The candidate models probed by McSNAC are composed of first-order reactions. We generated *in silico* data formed by ground-truth model GT1 given by Reaction (R2) at two different time points and fitted the candidate models (C1 to C7) shown in Table 1 to estimate reaction rates.


(R2) GT1:A+B→C

The McSNAC candidate models with the best fit *M* values describe few abundances at *t*_2_ reasonably well, but over- or under- shot values for certain species. For example, candidate model C1 (*B*→*C*) matches mean abundances of *B* but overestimates the mean abundance of *C* for model GT1 at *t*_2_ (details in the Supplementary Material). This behavior is expected as McSNAC uses first-order reactions to approximate second-order reactions in the ground-truth model. Next, we evaluated how the candidate models in McSNAC perform for predicting outcomes of *in silico* perturbation experiments where increased abundances of species (*e.g*., *B*) at *t*_1_ and assessed the ability of the candidate models to predict changes in the abundances of all the species at a later time *t*_2_. We defined a variable *Δμ*_*X*_ (*t*_1_*,t*_2_) describing changes in mean abundances of species X between two successive times *t*_1_ and *t*_2_ (>*t*_1_) for quantifying comparisons between the ground-truth and candidate models in the perturbation experiments. *Δμ*_*X*_ (*t*_1_*,t*_2_) is given by

(8) $$\Delta \mu_{X}\left(t_{1},t_{2}\right)=\mu_{X}\left(t_{2}\right)-\mu_{X}\left(t_{1}\right)$$

**Figure 2.**
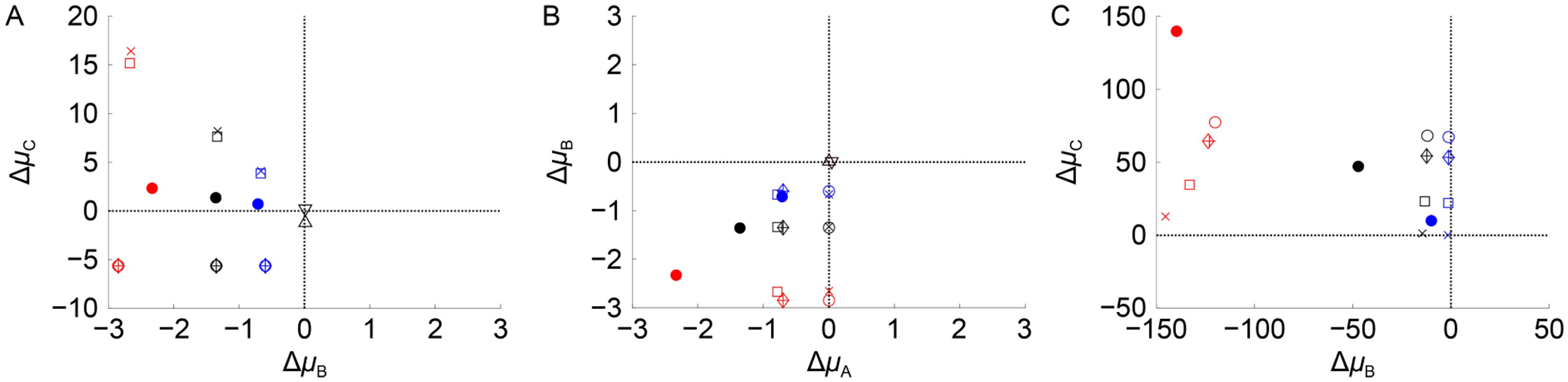
McSNAC is capable of predicting the effects of perturbations in simple nonlinear systems. (A) Predictions of the change in averages through time in *B* and *C* resulting from perturbations of *B* for the ground-truth model indicated by Reaction GT1, as predicted by the approximations in Models C1-C7. Large, filled circles represent the ground-truth, and markers for candidate predictions are as follows. C1: *B* → *C* (X’s); C2: *A* → *C* (Squares); C3: *B* ↔ *C* (Open Circles); C4: *A* ↔ *C*, *B* → *C* (Diamonds); C5: *A* ↔ *C*, *B* ↔ *C* (+’s); C6: *C* → *B* (upward-facing triangles); and C7: *C* → *A*, *C* → *B* (downward-facing triangles). Black points are estimations without a perturbation, red points are estimations in which *B* was perturbed upwards two-fold, and blue points are estimations in which *B* was perturbed downwards two-fold. *Δμ*_*B*_ and *Δμ*_*C*_ are defined as the change in averages through time, shown in Eq. (8). (B) Predictions of the change in averages through time in *A* and *B* resulting from perturbations of *B* for the ground-truth model indicated by Reaction GT1. Markers and perturbation information are the same as in (A). (C) Predictions of the change in averages through time resulting from perturbations of *B* for the ground-truth model indicated by Reaction GT2, as predicted by the approximations in Models C1−C5. Markers are the same as in (A), but perturbations of *B* are 10-fold.

*Δμ*_*X*_ (*t*_1_*,t*_2_) > 0 (or < 0) indicates net production (or consumption) in the mean abundance of X as the kinetics progress from time *t*_1_ to *t*_2_. To illustrate how *Δμ*_*X*_ (*t*_1_*,t*_2_) can be used to analyze outcomes of a perturbation experiment, if *Δμ*_*C*_ (*t*_1_*,t*_2_) > 0 in the ground-truth model and also in a candidate model (*e.g*., C1) when abundance of *B* is increased at time *t*_1_, it implies that candidate model C1 is able to qualitatively predict the outcome of the perturbation. The quantitative difference between the ground-truth model and the prediction made by the candidate model can be given by the squared distance between *Δμ*_*X*_ (*t*_1_*,t*_2_) obtained from the ground-truth and a candidate model. First, we investigated results from increasing or decreasing abundances of *B* at time *t*_1_ in the ground-truth model GT1 and the candidate models (Table 1, Fig. 2A and Supplementary Fig. S2). The candidate models C1 and C2 correctly predict the net production of species *B* and *C* or *Δμ*_*B*_ (*t*_1_*,t*_2_) > 0 and *Δμ*_*C*_ (*t*_1_*,t*_2_) > 0 as in GT1. In addition, as abundance of *B* is increased (or decreased) in the perturbation experiment the productions of *B* and *C* are increased (or decreased); this monotonic behavior is also correctly predicted by candidate models C1 and C2. However, the values of *Δμ*_*B*_ (*t*_1_*,t*_2_) and *Δμ*_*C*_ (*t*_1_*,t*_2_) in the models C1 and C2 are different than that in the ground-truth model GT1 indicating that the candidate models are able to correctly predict outcomes of perturbations qualitatively but not quantitatively (Table 1 and Fig. 2A). The models *C*3 and *C*7 are unable to correctly predict the above qualitative changes correctly (Table 1 and Fig. 2A). However, none of the candidate models are able to capture the decrease (or increase) in *Δμ*_*A*_ (*t*_1_*,t*_2_) as abundance of *B* is increased (or decreased) in GT1 (Fig. 2B) as the first-order reactions are unable to capture the dependencies of consumption of *A* and *B* given by the second-order reaction in GT1. Thus, as long as the directionality of the reaction, *i.e*., irreversible production of *C* from *B* and *A* is correctly captured in the candidate models they are able to qualitatively predict outcomes of the perturbation for most of the species.

To extend the exploration of approximating simple nonlinear dynamics, we simulated data from a similar nonlinear system shown by Reaction (R3).


(R3) GT2:A+B↔C

Because the directionality of this model depends on the relative abundance of reactants and products, we formed three sets of data: (1) reactants are more abundant than product (rightward directionality); (2) product is more abundant than reactants (leftward directionality); (3) reactants and product are approximately equal in magnitude (almost no directionality). We modeled each set of data formed by this nonlinear system with the approximations in candidate models C1−C5. We show that for the first case (rightward directionality) we can qualitatively capture the predicted effects of a perturbation of *B* (Fig. 2C). However, perturbations of *A* predicted larger effects in *Δμ*_*C*_ for these data than for GT1 (Supplementary Fig. S3). An important note is that perturbations of *C* again generated no impact on either *Δμ*_*A*_ or *Δμ*_*B*_, which is not consistent with the bidirectional nature of GT2. This could be explained by the net forward directionality of the data construction, where a net production of *C* occurs when no perturbation occurs. A more complete description of the results of models C1−C5 on each dataset is provided in Supplementary Table S1.

### McSNAC is capable of predicting effects of perturbations in complex non-linear signaling networks

**Figure 3.**
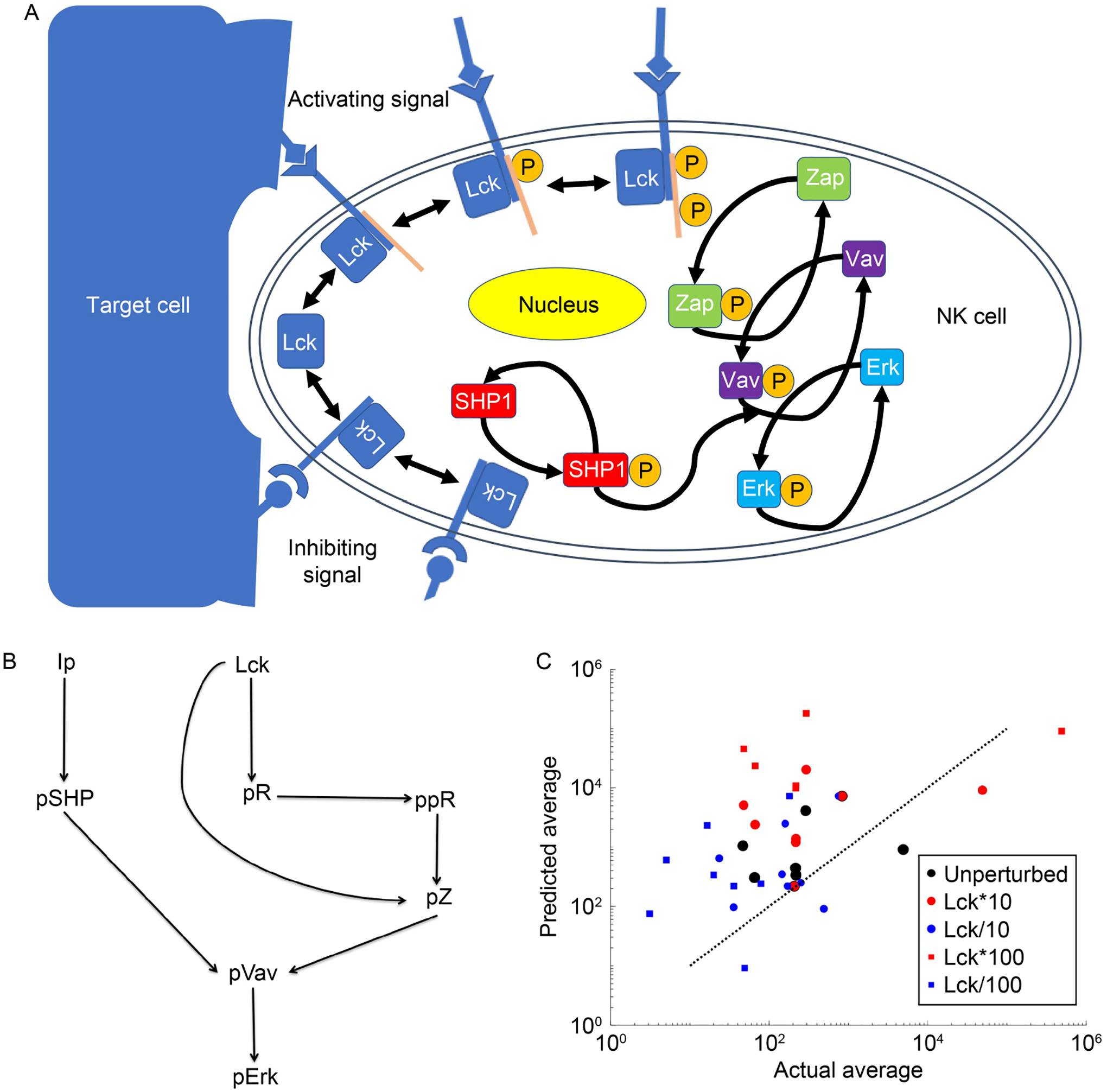
McSNAC is capable of predicting the effects of perturbations in complicated nonlinear systems. (A) A representation of the ground-truth system used for the *in silico* nonlinear signaling network simulation of Erk signaling in NK cells [22,23]. NK cells recognize ligands corresponding to activating and inhibiting signals on target cells. These signals result in binding of various proteins to the receptor, activation (phosphorylation) of proteins, and a cascade of phosphorylation to proteins in the cytosol of the NK cell. The proteins Erk and Vav become phosphorylated, which in turn mediate lysis of target cells. (B) A first-order network representation of (A), passed to McSNAC for estimation of kinetic parameters. (C) The results of perturbations performed on activated Lck. Actual averages of each species resulting from Lck perturbation in (A) are compared with predicted ones from the first-order approximation in (B).

Here we applied McSNAC to test its ability to predict effects of changes in species abundances for a signaling model describing membrane proximal signaling events in an NK cell. The signaling reaction contains multiple binding-unbinding and enzymatic reactions—all of which are composed of coupled second-order reactions. The NK cell signaling model, which is the ground-truth model here, is described in Fig. 3A. The model contains 18 molecule types, 112 total species (molecules and complexes of bound molecules), and 53 reactions, and is simulated using BioNetGen, a software package that specializes in simulating biological reaction networks [16,17]. We set up a candidate model, where we assumed a subset of protein species are measured in cytometry experiments, and these proteins react via first-order reactions as shown in Fig. 3B. Because this model only explores a subset of the total number of species and omits measurements such as un-phosphorylated proteins, it is reflective of a CyTOF experiment studying signaling kinetics where many proteins are unmeasured. Omission of unmeasured proteins does not impact a simpler ground-truth linear system (Supplementary Fig. S4), so we continue to this more complex nonlinear ground-truth simulation. First, we simulated kinetics of signaling protein abundances in single cells in the ground-truth model using BioNetGen over a time interval *t* = 0 s to 60 s. The details regarding rate constants and initial distributions of protein abundances are given in Materials and Methods section and the supplemental BioNetGen file. The mean abundances and covariances calculated for the protein species in the candidate model were then fitted at two time points (*t*_1_ = 0 s and *t*_2_ = 30 s) to estimate rates in the matrix ***M***. The best fit value of ***M*** estimated mean abundances well for several species, however, over- and under- estimated for 4 out of 8 species (Fig. 3C). This behavior is expected as McSNAC used first-order reactions to model a ground-truth model containing nonlinear reactions. Next, we evaluated the ability of the candidate model to describe outcomes of perturbations of total abundances for specific protein species. We increased and decreased the total amount of the Src kinase Lck at time *t*_1_ = 0 in the ground-truth model and the candidate model with the best fit ***M*** and compared mean abundances at a later time *t*_2_ = 30 s (Fig. 3C). Lck is a kinase that regulates early time signaling events such as receptor tyrosine phosphorylation and directly and indirectly influence abundances of the species considered in the candidate model. We found that mean protein abundances at *t*_2_ = 30 s in the candidate model showed a statistically significant positive correlation (= 0.33) with that in the ground-truth model (Fig. 3C), thus, the predictions for Lck perturbation in McSNAC are able to qualitatively capture the changes in mean abundances in the ground-truth model.

**Figure 4.**
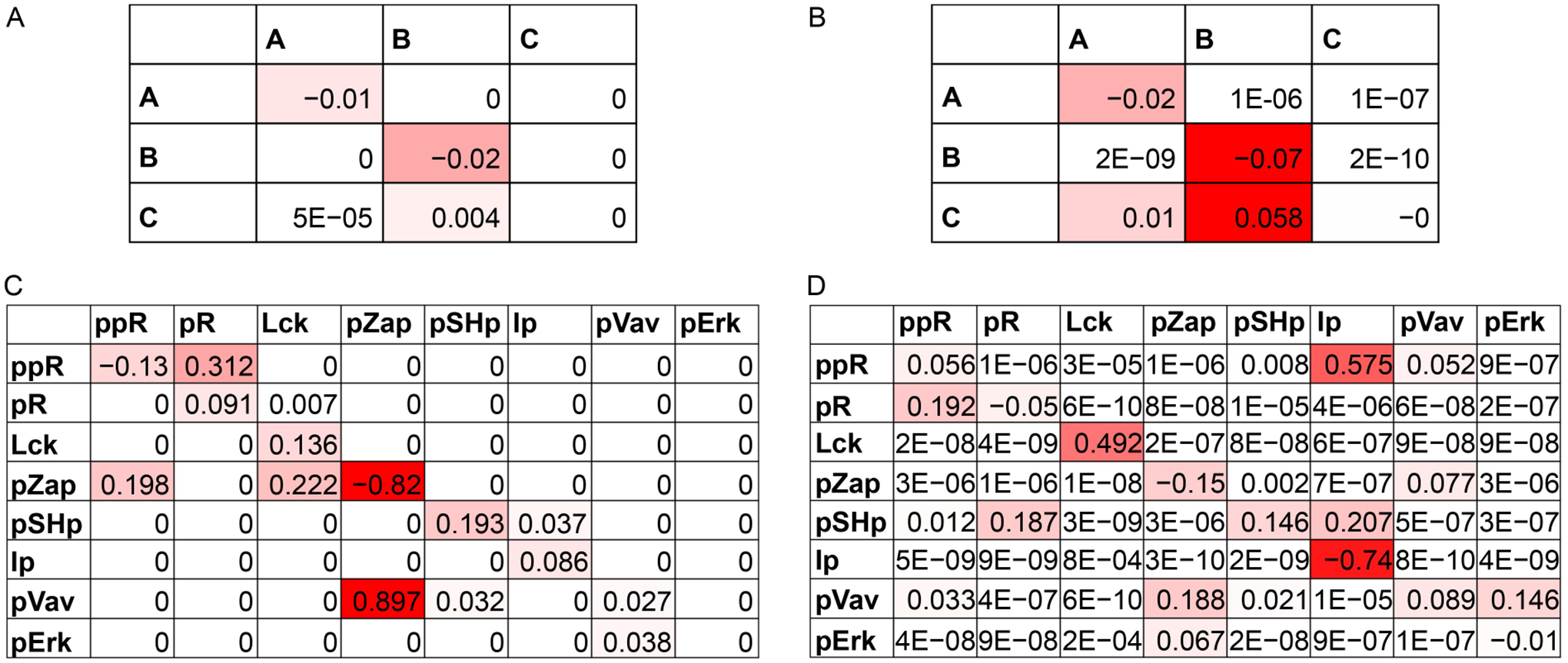
McSNAC displays mixed results when modeling non-linear networks without a priori network proposal. (A–B) Shows elements of the *M* matrix when data generated from the nonlinear network GT1 are modeled with a linear network given by (A) model C2 in Table 1, and with (B) a *fully connected* network. (C–D) Estimated elements of the *M* matrix when data generated from the *in silico* network in Fig. 3A are modeled with (C) the proposed linear network in Fig. 3B, and with (D) a *fully connected* network. The shades of red show the magnitude of the value of each estimated element.

Above we constructed the candidate linear model by intuition, therefore, we investigated if such a candidate model can be constructed using a *fully connected* linear model where any pair of species interact with each other. We reasoned based on our results (Fig. 1D) for the linear model that estimated rates over a user defined threshold in the fully connected model can point to a candidate model. We found that when data are generated from a simple nonlinear model (GT1), parameter estimations in the fully connected model resembled a linear candidate model C2 in Table 2 (Fig. 4A–B). However, for data generated from a more complex nonlinear model in Fig. 3A, reactions in fully connected network associated with larger values of estimated values showed similarities and dissimilarities with our candidate model in Fig. 3B (Fig. 4C–D). The fully connected network for our *in silico* data did correctly reconstruct many of the existing signaling steps, such as the signal from the inhibitory receptor to pSHP, from the single-phosphorylated activating receptor to the double-phosphorylated activating receptor, and from pZap70 to pVav1. However, there were some notable deviations. For instance, in the fully connected network there is a large kinetic rate for the reaction pZap → pErk, with a small rate for pVav → pErk, which skips a signaling reaction (*e.g*., pVav induced phosphorylation of Erk) in the ground-truth model (Fig. 4C–D). Additionally, the inhibitory receptor is found to phosphorylate ITAMs associated with activating receptor, another step which is not realistic. These unrealistic estimations could arise due to the inability of the linear network to correctly capture co-dependencies between the species in a complex non-linear ground-truth model. Therefore, we recommend using the linear representation as a generation tool.

### Graphical User Interface

**Figure 5.**
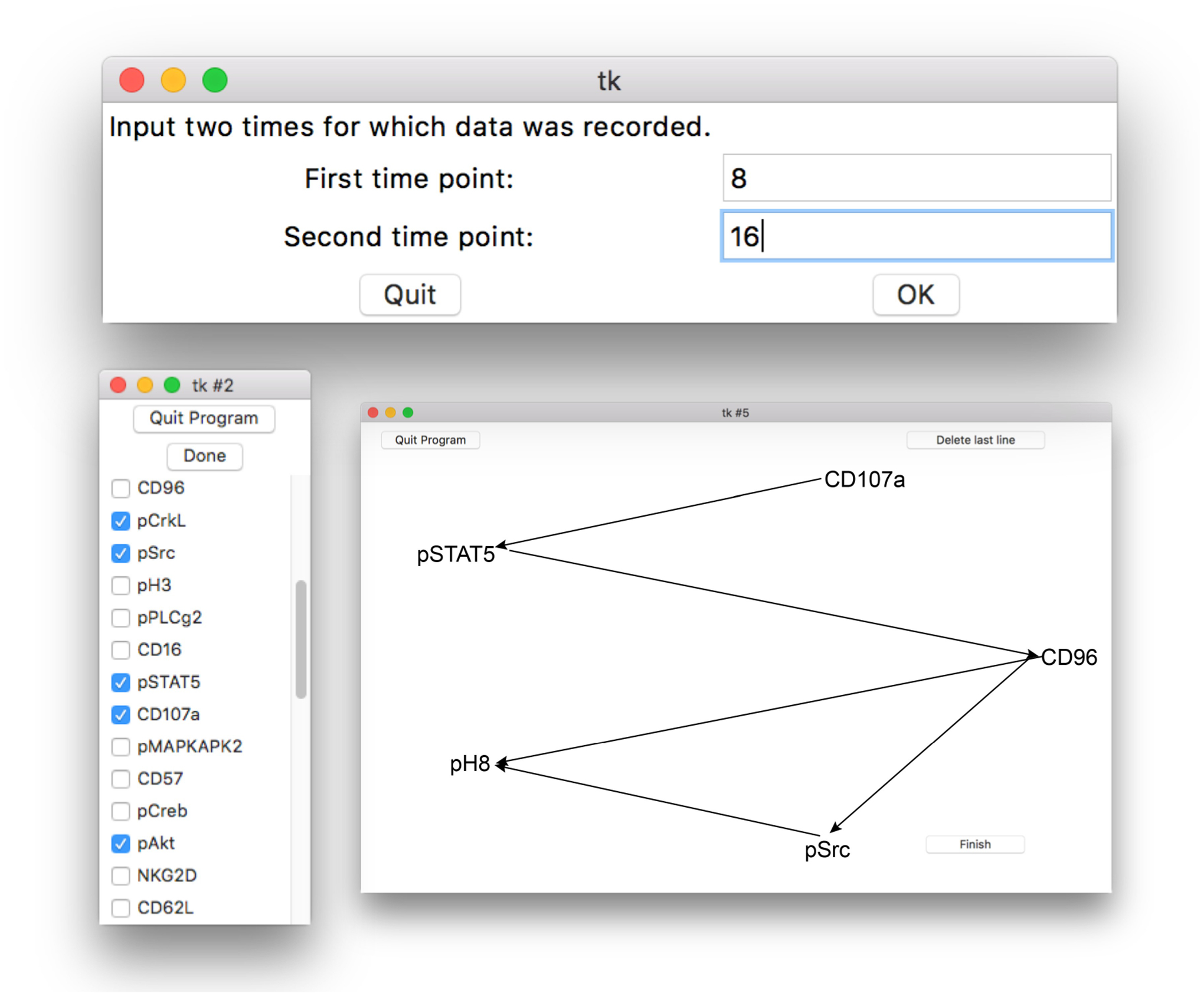
McSNAC is designed for ease-of-use with an intuitive graphical user interface. The software starts by having a user drag a file to the terminal and then following on-screen prompts to indicate the files they would like to analyze, the time difference between data collection, and the proteins to include in the network. Selected proteins are displayed in a circle and the user can draw arrows connecting proteins to indicate the proposed model network. Upon completion, the network is displayed to the user with flux calculations for each connection, which are displayed by hovering over the arrow of interest.

McSNAC is designed with ease-of-use in mind. It can be run by dragging a file to a terminal and following on-screen prompts to enter information about the desired signaling network to model (Fig. 5). The output of the software is an interactive display which shows the flux arising from each connection (calculated by *k*_*i*→*j*_*μ*_*i*_ (*t*_1_)). Additionally, a .csv file is generated to save the results for later viewing. This intuitive graphical user interface (GUI) and output should make McSNAC approachable for biologists not accustomed to running computational software from the command line.

## DISCUSSION

McSNAC is a scalable software package for developing signaling kinetic models based on first-order biochemical reactions using time-stamped CyTOF data. The estimation of kinetic rates in McSNAC accounts for cell-cell variations of protein abundances found in cytometry data and separates changes in protein phosphorylation generated due to tonic or basal signaling and signaling induced by receptor stimulation. Our previous worked showed that the framework based on first-order reaction kinetics is able to describe synergy between cytokine treatment and receptor stimulation for CyTOF data obtained for primary human NK cells. The McSNAC software is based on this framework and implements a GUI to add features such as protein species selection and specification of a reaction of interest as desired by users.

Application of McSNAC for a set of *in silico* experiments showed the framework based on first-order reactions is able to accurately estimate rates when the proposed reaction network contains the wiring of the ground-truth model consisting of first-order reactions. Candidate networks that do not contain the wiring of the ground-truth model produce substantially large values of the cost function. Our *in silico* tests showed that when ground-truth models are nonlinear second-order biochemical reactions, McSNAC is able to generate qualitatively correct predictions for perturbations of protein abundances when directionality of reactions are correctly accounted for in McSNAC. We also found these agreements with qualitative predictions hold when a subset of signaling species is measured and modeled in McSNAC for synthetic cytometry experiments. Perturbations of cell signaling are commonly carried out to analyze signaling networks. Therefore, McSNAC can be a valuable tool for screening potential models that can be hypothesized for describing cytometry datasets.

An important limitation of the first-order approximation is its inability to capture non-monotonic behavior. In many true signaling networks, activity is differentially regulated at various time intervals. For instance, several proteins (*e.g*., pAkt) display non-monotonic response—such behaviors can arise as protein degradation initiated by early signaling events can decrease abundances of phosphorylated forms of proteins. The first-order framework is unable to capture such non-monotonic temporal changes. However, one can divide the non-monotonic kinetics into time intervals where the kinetics is monotonic and use McSNAC to model each of these intervals separately. The rate parameters obtained from those piece-wise models could provide insight regarding change of specific signaling reactions with time. McSNAC is also not able to fit many species abundances well, and generate correct predictions for protein abundances at future times when the underlying model contains non-linear biochemical reactions. Conceptually, McSNAC is capable of fitting data at more than two timepoints. Incorporating more data is likely to improve parameter estimation for a ground-truth linear signaling network [18,19], but more realistic non-linear signaling networks suffer as the first-order approximation will change at varying times. Improving on these limitations will require including non-linear reactions into the framework, a task that will be computationally demanding, as matrix exponentiation must be abandoned for numerical ODE solutions.

The GUI and code base will be updated to reflect features desired by users. Some possible future directions would be to implement a method to automatically report confidence intervals on parameter estimates, implementing the ability to run on high-performance computing clusters, or adapting it to other data types other than .fcs files. The software is designed exclusively for OS X operating systems, but the recent addition of the Windows PowerShell may make it feasible to adapt it for Windows as well. The software is intended to be open-source, and we encourage users to request features they’d like to see and/or implement them themselves. The simulated annealing scheme is written in Fortran, and the GUI and parent script are written in Python. The software can be found on github (dweth/mcsnac), and code edits can be made there as well.

## MATERIALS AND METHODS

### Solution of Eq. (2)

In matrix notation where *x* = {*x*_i_} is a column vector, the ODE in Eq. (2) can be rewritten as

$$\frac{dx}{dt}=Mx$$

If ***S*** denotes the similarity transformation matrix that diagonalizes ***M*** to a diagonal matrix ***D*** then,

$$D=SMS^{-1}$$

***D*** contains eigenvalues {*λ*^(i)^} of ***M*** as diagonal elements. Defining *y* = ***D****x*, the ODE in *x* can be rewritten as

$$\frac{dy}{dt}=SMS^{-1}y=Dy.$$

Since ***D*** is a diagonal matrix, the above equation represents a set of uncoupled equations in *y*_i_, where *y* = {*y*_i_}. The equation in *y*_i_ is given by, *dy*_*i*_*/dt* = *λ*^*(i)*^*y*_*i*_; therefore, *y*_i_(*t*) = *y*_i_(0)exp(*λ*^(i)^*t*) or *y*(*t*) = exp(***D****t*)*y*(0). Since, *x* = ***S***^−1^*y*, *x*(*t*) is given by, *x*(*t*) = ***S***^−1^*y*(*t*) = ***S***^−1^exp(***D****t*)***S S***^−1^ *y*(0) = [***S***^−1^exp(***D****t*) ***S***]*x*(0) = exp(***S***^−1^(***D****t***)*S***) *x*(0) = exp(***M***t)*x*(0).

### Generation of single cell *in silico* data for first-order and nonlinear reaction models

First-order *in silico* networks were simulated with MATLAB. For the results shown in Fig. 1A–B, 2,500 cells were assigned initial (*t*_1_ = 0) values of *n* protein abundances drawn from lognormal distributions and the single cell kinetics were simulated using the closed form solution of Eq. (2). Averages and covariances at *t*_2_ (= 30 s) were calculated by the software and used for fitting. For the results shown in Fig. 1C, means and covariances at *t*_2_ used for fitting were simply calculated from the closed-form solutions given in Eqs. (5)–(6).

Nonlinear reaction networks in ground-truth models GT1, GT2, and the NK cell signaling model (Fig. 3A) were simulated using the software BioNetGen. Protein abundances in 250 single cells at *t*_1_ = 0 were drawn from lognormal distributions. The rate constants and the parameters for the lognormal distributions are provided in BioNetGen scripts shown in the Supplementary Material. Mean abundances and covariances at a later time *t*_2_ (= 30 s) were calculated in McSNAC from the synthetic single cell data. Perturbations of abundances of A, B, and C for Fig. 2A–B, and Supplementary Fig. S2 were generated by multiplying the initial concentration of the given protein by 2 or 1/2, while Fig. 2C and Supplementary Fig. S3 were generated by multiplying the initial concentration of the given protein by 10 or 1/10.

Initial abundances for the 8 measured phospho-proteins, their 7 unmeasured unphosphorylated counter-parts (activating receptor is measured as both phosphorylated and double-phosphorylated), activating and inhibiting ligand, and a pErk phosphatase were sampled from lognormal distributions for the BioNetGen simulation of the NK cell signaling model. These initial values for all 250 cells can be found in the Supplementary Materials (x1_data.txt). For Lck perturbation experiments, the initial abundance of phosphorylated and unphosphorylated Lck across all cells for both was multiplied by the constant indicated in Fig. 3C.

### Generation of first-order ground-truth model and candidate models

For Fig. 1D, we constructed an *in silico* dataset based on the reaction network in Reaction (R4), a representation of Reaction (R1) with *p* = 4.


(R4) $$X_{1}\overset{k_{1\to 2}}{\to }X_{2}\overset{k_{2\to 3}}{\to }X_{3}\overset{k_{3\to 4}}{\to }X_{4}$$

We constructed a Boolean reaction matrix ***A*** where presence (or absence) of a reaction *j*→ *i* is indicated by 1 (or 0) at the *ij* th element. The ground-truth model given by Reaction (R4) is given by the matrix ***A***_ground-truth_ below:

$$A_{\text{ground-truth}}=\begin{array}{cccc} 0 & 0 & 0 & 0\\ 1 & 0 & 0 & 0\\ 0 & 1 & 0 & 0\\ 0 & 0 & 1 & 0 \end{array}$$

Since there are 12 off diagonal elements in the above matrix, and we construct 2^12^ candidate models where the each of the 12 off diagonal elements are set to 0 or 1. The candidate models that contain non-zero values in ***A*** corresponding to *k*_1→2_, *k*_2→3_, and *k*_3→4_ contain the ground-truth model in Reaction (R4). The “number of true connections” is given by

$$n_{-}\text{true}\_\text{onnections}=\sum_{i,j}\left({\left[A_{\text{ground-truth}}\right]}_{ij}AND{\left[A_{\text{proposed}}\right]}_{ij}\right)$$

The variable *n_true_connections* = 3 whenever a candidate model includes the ground-truth reaction in (R4).

### Estimation of parameter confidence intervals

We estimate confidence intervals (CIs) for the elements of the ***M*** matrix following a Profile Likelihood based approach [20]. First, we estimate the ***M*** matrix corresponding to the minimum of the cost function in Eq. (7) using simulated annealing. Then we use this best fit value of the ***M*** matrix to generate a large number of samples (*e.g*.,100,000) of matrix M* following $M_{ij}^{*}=M_{ij}\times b^{a_{ij}}$, where *b* is a scalar (*e.g*., 2), and *a*_*ij*_ is a random number between −1 and 1 drawn from a uniform distribution U(−1,1). For each sample of M* we calculate a variable *d* = *χ*^2^ ({M*}) − *χ*^2^ ({*M*}), describing the difference in the cost function χ^2^ defined in Eq. (8) between the best fit ***M*** and the sampled M*. Now, we chose a specific element (*e.g., k*,*l*) of the ***M*** matrix and create a list of the *a*_*kl*_ values that were used to generate M*_kl_, and bin the *a*_*kl*_ values between −1 to +1. Next, *d* values that correspond to the sampled {M*_kl_} and specific bins of *a*_*kl*_ are collected, thus, each bin of *a*_*kl*_ would contain multiple values of *d*. We determine the smallest value of *d* for each bin in the *a*_*kl*_ space. Then, starting from the bin that contains *a*_*kl*_ = 0 and advancing to the bins of *a*_*kl*_ > 0 (or *a*_*kl*_ < 0) we determine the first bin that generates a minimum *d* ≥ 2.71, this first bin corresponds to the upper (or lower) bound of *M*_*kl*_.

It is important to note that this method suffers the curse of dimensionality. As the number of dimensions increases, so too must the number of samples to account for coverage of all bins in all dimensions. We report confidence intervals for our 13-dimension parameter benchmarking (Fig. 1A), but not the 40-dimensional one (Fig. 1B), because we do not have the computational capability to form that many samples. This is a limitation of our confidence-interval estimation technique in high dimensions.

### Simulated annealing hyperparameters

Simulated annealing (SA) is a global optimization algorithm that searches for an optimum value of a cost function, *χ*^2^. The optimization is based on the Metropolis algorithm for simulating a thermodynamic system at temperature T where system variables (*e.g*., positions of atoms) associated with an energy *E* are distributed in a Boltzmann distribution (∝exp(−*E*/(*k*_*B*_*T*))). *k*_*B*_ is the Boltzmann constant. Slowly cooling or annealing the system takes the system to lower energy states (*e.g*., crystals) from higher energy random configuration of system variables (*e.g*., gaseous states). This approach can be adapted for parameter estimation where the ‘energy’ *E*(= *χ*^2^) represents the cost function which is a function of the parameter values. In SA the temperature parameter (*k*_*B*_*T*) is initiated at larger values (*e.g*., comparable to the largest values of *E*) and is decreased slowly to low values. As the temperature parameter is decreased gradually the system is supposed to relax to parameter configuration corresponding to the global minimum of *E*. However, the landscape of *E* can contain multiple minima and attaining the global minimum is not guaranteed in such situations. Several improvements on the basic SA algorithm have been proposed to deal with such scenarios. At each value of the temperature parameter, the Metropolis algorithm is run for sufficiently large number of Monte Carlo trials to make sure equilibrium Boltzmann distributions are realized in the simulations. At high temperatures, large increases in cost function can be accepted, while at low temperatures, only insignificant increases will be accepted. Simulated annealing gradually lowers the temperature parameter according to a cooling rate until a ‘global’ minimum is reached. For more information on simulated annealing, refer to [21].

All simulations reported in the manuscript used the same hyperparameters (cooling rate, number of Monte Carlo samples, *etc*.) found in the Supplementary Material script Simulated_Annealing.f, with the exception of the number of iterations of the temperature cooling loop. Table 2 below shows the number of steps used for each figure.

## Supplementary Material

supplementary 1

supplementary 2

## Figures and Tables

**Table 1:** List of candidates first-order models for describing a unidirectional second-order reaction A + B → C in ground-truth model GT1

Candidate models	Directionality included	Prediction of perturbation outcome for changing *B* abundance
*C*1 : *B* → *C*	Y	*Δμ*_*B*_ (*t*_1_,*t*_2_) and *Δμ*_*C*_ (*t*_1_,*t*_2_) are qualitatively accurate.
*C*2: *A* → *C*, *B* → *C*	Y	Same as above, however, does not capture the behavior in *Δμ*_*A*_ (*t*_1_,*t*_2_).
*C*3 : *B* ↔ *C*	Y	Qualitatively accurate for *Δμ*_*B*_ (*t*_1_,*t*_2_) but not for *Δμ*_*C*_ (*t*_1_,*t*_2_).
*C*4: *A* ↔ *C*, *B* → *C*	Y	Same as above
*C*5 : *A* ↔ *C*, *B* ↔ *C*	Y	Same as above
*C*6 : *C* → *B*	N	Inaccurate for both *Δμ*_*B*_ (*t*_1_,*t*_2_) and *Δμ*_*C*_ (*t*_1_,*t*_2_) (≅ 0)
*C*7 : *C* → *A*, *C* → *B*	N	Same as above

**Table 2:** Number of cooling steps for simulated annealing for results shown in each figure

Figure number	Number of cooling steps in simulated annealing
1A, 1B	2,000
1C	3,000
2A (same as S2)	2,000
2B (same as S3)	2,000
3C (same as S1)	5,000
S4	5,000
